# The Relations of Bacteria to Practical Surgery

**Published:** 1891-05

**Authors:** John B. Roberts

**Affiliations:** Professor of Surgery in the Woman's Medical College of Pennsylvania, Professor of Anatomy and Surgery in the Philadelphia Polyclinic


### The Relations of Bacteria to Practical Surgery.

BY JOHN B. ROBERTS, M.D.

Professor of Surgery in the Woman's Medical College of Pennsylvania, Professor of Anatomy and Surgery in the Philadelphia Polyclinic.

The revolution which has occurred in practical surgery since the discovery of the relation of micro-organisms to the complications occurring in wounds has caused me to take up this subject for discussion. Although many of my hearers are familiar with the germ theory of disease, it is possible that it may interest some to have put before them, in a short address, a few points in bacteriology, which are of value to the practical surgeon.

It must be remembered that groups of symptoms which were formerly classed under the heads of "inflammatory fever," "symptomatic fever," "traumatic fever," "hectic fever," and similar terms, varying in name with the surgeon using them, or with the location of the disease, are now known to be due to the invasion of the wound by microscopic plants. These bacteria, after entering the blood current at the wound, multiply with such prodigious rapidity that the whole system gives evidence of their existence. Suppuration of wounds is undoubtedly due to these organisms, as is tubercular disease, whether of a surgical or medical character. Tetanus, erysipelas and many other surgical conditions have been almost proved to be the result of infection by similar microscopic plants, which, though acting in the same way, have various forms and life-histories.

A distinction must be made between the "yeast plants," one of which produces thrush, and the "mould plants," the existence of which as parasites in the skin gives rise to certain cutaneous diseases. These two classes of vegetable parasites are foreign to the present topic, which is surgery; and I shall, therefore, confine my remarks to that groupe of vegetable parasites to which the term bacteria has been given. These are the micro-organisms whose actions and methods of growth particularly concern the surgeon. The individual plants are so minute that it takes in the neighborhood of ten or fifteen hundred of them grouped together to cover a spot as large as the full stop or period used in punctuating an ordinary newspaper. This rough estimate applies to the globular or egg-shaped bacteria to which is given the name "coccus" (plural cocci). The cane, or rod-shaped bacteria are rather large plants. Fifteen hundred of these placed end to end would reach across the head of a pin. Because of the resemblance of these latter to a walking stick they have been termed bacillus (plural bacilli).

The bacteria most interesting to the surgeon belong to the cocci and the bacilli. There are other forms which bacteriologists have dubbed with similar descriptive names, but they are more interesting to the physician than to the surgeon. Many micro-organisms, whether cocci, bacilli, or of other shapes, are harmless; hence they are called non-pathogenic, to distinguish them from the disease-producing, or pathogenic germs.

As many trees have the same shape and a similar method of growing, but bear different fruit; in the one case edible, and in the other poisonous—so, too, bacteria may look alike to the microscopist's eye, and grow much in the same way, but one will cause no disease, while the other will produce serious disease, such, perhaps, as tuberculosis of the lungs or brain.

Many scores of bacteria have been by patient study, differentiated from their fellows and given distinctive names. Their nomenclature corresponds in classification and arrangement with the nomenclature adopted in different departments of botany. Thus, we have the pus-causing chain coccus (streptococcus pyogenes), so called because it is globular in shape; because it grows with the individual plants attached to each other, or arranged in a row like a chain of beads upon a string; and because it produces pus. In a similar way we have the pus-causing grape-coccus of a golden color (staphylococcus pyogenes aureus). It produces pus, grows with the individual plants arranged somewhat after the manner of a bunch of grapes, and when millions of them are collected together the mass has a golden yellow hue. Again, we have the bacillus tuberculosis; the rod-shaped plant which is known to cause tuberculosis of the lungs, joints, brain, etc.

It is hardly astonishing that these fruitful sources of disease have so long remained undetected, when their microscopic size is borne in mind. That some of them do cause disease is undisputed, since bacteriologists have, by their watchful and careful methods, separated a single plant from its surroundings and congeners, planted it free from all contamination and observed it produce an infinitesimal brood of its own kind. Animals and patients inoculated with the plants thus cultivated have rapidly become subjects of the special disease which the particular plant was supposed to produce.

The difficulty of such investigations becomes apparent when it is remembered that under the microscope many of these forms of vegetable life are identical in appearance, and it is only by observing their growth, when in proper soil, that they can be distinguished from each other. In certain cases it is quite difficult to distinguish them by their physical appearances produced during their growth. In such instances it is only after an animal has been inoculated with them that the individual parasite can be accurately recognized and called by name. It is known by the results it is capable of producing.

The various forms of bacteria are recognized, as I have said, by their method of growth, and by their shape. Another means of recognition is their individual peculiarity of taking certain dyes so that special plants can be recognized, under the microscope, by the color given to them, which they refuse to give up when treated with chemical substances which remove the stains from, or bleach, all the other tissues which at first have been similarly stained.

The similarity between bacteria and the ordinary plants with which florists are familiar is indeed remarkable. Bacteria grow in animal and other albuminous fluids, as a soil, but it is just as essential for them to have a suitable soil as it is for the corn or wheat that the farmer plants in his field. By altering the character of the albuminous fluid in which the micro-organism finds its subsistence, these small plants can be given a vigorous growth or may be actually starved to death. The farmer knows that it is impossible for him to grow the same crop year after year in the same field, and he is, therefore, compelled to rotate his crops. So it is with these microscopic plants which we are considering. After a time the culture fluid or soil becomes so exhausted of its needed consitituents by the immense number of plants living in it that it is unfit for their life and development. Then this particular plant will no longer thrive, but some other form of bacterium may find in it the properties required for functional activity, and grow vigorously. It is probable that exhaustion or absence of proper soil is an important agent in protecting man from sickness due to infection from bacteria. The ever present bacteria often gains access to man's blood through external wounds, or through the lungs and digestive tracts; but unless a soil suited for their development is found in his fluids, the plants will not grow. If they do not grow and increase in numbers they can do little harm.

Again, there are certain bacteria which are so antagonistic to each other that it is impossible to make them grow in company, or to co-exist in the blood of the same individual. For example, an animal inoculated with erysipelas germs cannot be successfully inoculated immediately afterward with the germs of malignant pustule. Such antagonism is illustrated by the impossibility of having a good crop of grain in a field overrun with dasies. On the other hand, however, there are some micro-organisms which flourish luxuriantly when planted together in the same fluid, somewhat after the manner of pumpkins and Indian corn growing between the same fence rails. Others seem unwilling to grow alone, and only flourish when planted along with some other germs. It is very evident, therefore, that bacteriology is a branch of botany, and that Nature shows the same tendencies in these minute plants as it does in the larger vegetable world visible to our unaided eyes.

As the horticulturist is able to alter the character of his plants by changing the circumstances under which they live, so can the bacteriologist change the vital processes and activities of bacteria by chemical and other manipulations of the culture substances in which the organisms grow. The power of bacteria to cause pathological changes thus may be weakened and attenuated; in other words, their functional power for evil is taken from them by alterations in the soil. The pathogenic, or disease-producing power, may be increased by similar, though not identical, alterations. The rapidity of their multiplication may be accelerated, or they may be compelled to lie dormant and inactive for a time; and on the other hand, by exhausting the constituents of the soil upon which they depend for life, they may be killed.

It is a most curious fact also that it is possible, by selecting and cultivating only the lighter colored specimens of a certain purple bacterium, for the bacteriologist to finally obtain a plant which is nearly white, but which has the essential characteristics of the original purple fungus. In this we see the same power which the florist has to alter the color of the petals of his flowers by various methods of selective breeding.

The destruction of bacteria by means of heat and antiseptics is the essence of modern surgery. It is, then, by preventing access of these parasitic plants to the human organism (aseptic surgery) or the destruction of them by chemical agents and heat (antiseptic surgery), that we are enabled to invade, by operative attacks, regions of the body which a few years ago were sacred.

When the disease-producing bacteria gain access to the tissues and blood of human and other animals by means of wounds, or through an inflamed pulmonary or alimentary mucous membrane, they produce pathological effects provided there is not sufficient resistance and health power in the animal's tissues to successfully antagonize the deleterious influence of the invading parasitic fungus. It is the rapid multiplication of the germs, which furnishes a continuous irritation, that enables them to have such a disastrous effect upon the tissues of the animal. If the tissues had only the original dose of microbes to deal with, the warfare between health and disease would be less uncertain in outcome. Victory would usually be on the side of the tissues and health.

The immediate cause of the pathogenic influence is probably the chemical excretions which are given out by these microscopic germs. All plants and animals require a certain number of substances to be taken into their organisms for preservation of their vital activities. After these substances have been utilized there occurs an excretion of other chemical products. It is probably the excretions from the many millions of bacteria circulating in the blood which give rise to the disease characteristic of the fungus with which the animal has been infected. The condition called saparæmia, or septic intoxication, for example, is undoubtedly due to the entrance of the excretory products (ptomaines) of putrefaction-bacteria into the circulation. This can be proved by injecting into an animal a small portion of these products obtained from cultures of the germs of putrefaction. Characteristic symptoms will at once be exhibited.

Septicæmia is a similar condition due to the presence of the putrefactive organisms themselves, and hence of their products or ptomaines also in the blood. The rapidity of their multiplication in this albuminous soil, and the great amount of excretion from the numerous fungi, makes the condition more serious than sapræmia. Clinically, the two conditions occur together.

The rapidity with which the symptoms may arise after inoculation of small wounds with a very few germs will be apparent when it is stated that one parasitic plant of this kind may, by its rapidity of multiplication, give rise to fifteen or sixteen million individuals within twenty-four hours. The enormous increase which takes place within three or four days is almost incalculable. It has been estimated that a certain bacillus only about 1–1000 of an inch in length could, under favorable conditions, develop a brood of progeny in less than four days which would make a mass of fungi sufficient to fill all the oceans of the world if they all had a depth of one mile.

Bacteria are present everywhere. They exist in the water, earth, air, and within our respiratory and digestive tracts. Our skin is covered with millions of them, as is every article about us. They can circulate in the lymph and blood, and reach every tissue and part of our organisms by passing through the walls of the capillaries. Fortunately, they require certain conditions of temperature, moisture, air and organic food for existence, and for the preservation of their vital activities.

If their surroundings are too hot, too cold, or too dry, or if they are not supplied with a proper quantity or quality of food from the soil in which they are living, the bacterium becomes inactive, until the surrounding circumstances change, or die absolutely. The spores which finally become full-fledged bacteria, are able to stand a more unfavorable environment than the adult bacteria. Many spores and adults, however, perish. Each kind of bacterium requires its own special environment to permit it to grow and flourish. The frequency with which an unfavorable combination of circumstances occurs limits greatly the disease-producing power of the pathogenic bacteria.

Many bacteria, moreover, are harmless and do not produce disease even when present in the blood and tissues. Besides this the white blood cells are perpetually waging war against the bacteria in our bodies. They take the bacteria into their interiors and render them harmless by eating them up, so to speak; and they crowd together and form a wall of white blood cells around the place where the bacteria entered the tissues, thus forming a barrier to cut off the blood supply to the germs and perhaps to prevent them from entering the general blood current.

The war between the white blood cells and bacteria is a bitter one. Many bacteria are killed; but, on the other hand, the life of many blood cells is sacrificed by the bacteria poisoning them with ptomaines. The tissue cells, if healthy, offer great resistance to the attacks of the army of bacteria. Hence, if the white cells are vigorous and abundant at the site of the battle, defeat may come to the bacteria, and the patient suffers nothing from the attempt of these vegetable parasites to harm him. If, on the other hand, the tissues have a low resistive power, because of the general debility of the patient, or of a local debility of the tissues themselves, and the white cells be weakened and not abundant, the bacteria will gain the victory, get access to the general blood current and invade every portion of the animal's body. Thus, a general, or a local disease, will be caused; varying with the species of bacteria with which the patient has been infected, and the degree of resistance on the part of the tissues.

From what has been stated, it must be evident that the bacterial origin of disease depends upon the presence of a disease-producing fugus and a diminution of the normal or healthy tissue resistance to bacterial invasion. If there is no fungus present, the disease caused by such fungus cannot develop. If the fungus be present and the normal or health tissue-resistance be *undiminished*, it is probable that disease will not occur. As soon, however, as overwork, injury of a mechanical kind, or any other cause diminishes the local or the general resistance of the tissues and individual, the bacteria have the upper hand, and are able to produce their malign effect.

Many conditions favor the bacterial attack. The patient's tissue may have an inherited peculiarity, which renders it easy for the bacteria to find a good soil for development; an old injury or inflammation may render the tissues less resistant than usual; the point at which inoculation has occurred may have certain anatomical peculiarities which make it a good place in which bacteria may multiply; the blood may have undergone certain chemical changes which render it better soil than usual for the rapid growth of these parasitic plants.

The number of bacteria originally present makes a difference also. It is readily understood that the tissues and white blood cells would find it more difficult to repel the invasion of an army of a million microbes, than the attack of a squad of ten similar fungi. I have said that the experimenter can weaken and augment the virulence of bacteria by manipulating their surroundings in the laboratory. It is probable that such a change occurs in nature. If so, some bacteria are more virulent than others of the same species; some less virulent. A few of the less virulent disposition, would be more readily killed by the white cells and tissues than would a larger number of more virulent ones. At other times the danger from microbic infection is greater because there are two species introduced at the same time; and these two multiply more vigorously when together than when separated. They are, in fact, two allied hosts trying to destroy the blood cells and tissues. This occurs, for instance, when the bacteria of putrefaction and the bacteria of suppuration are introduced into the tissues at the same time. The former causes sapræmia and septicæmia, the latter cause suppuration. The bacteria of tuberculosis are said to act more viciously if accompanied by the bacteria of putrefaction. Osteomyelitis is of greater severity, it is believed, if due to a mixed infection with both the white and the golden grape-coccus of suppuration.

I have previously mentioned that the bacteria of malignant pustule are powerless to do harm when the germs of erysipelas are present in the tissues and blood. This is an example of the way in which one species of bacteria may actually aid the white cells, or leucocytes, and the tissues in repelling an invasion of disease-producing microbes.

Having occupied a portion of the time allotted to me in giving a crude and hurried account of the characteristics of bacteria, let me discuss the relations of bacteria to the diseases most frequently met with by the surgeon.

Mechanical irritations produce a very temporary and slight inflammation, which rapidly subsides; because of the tendency of nature to restore the parts to health. Even severe injuries, therefore, will soon become healed and cured, if no germs enter the wound.

Suppuration of operative and accidental wounds was, until recently, supposed to be essential. We now know, however, that wounds will not suppurate if kept perfectly free from one of the dozen forms of bacteria that are known to give rise to the formation of pus.

The doctrine of present surgical pathology is that suppuration will not take place, if pus-forming bacteria are kept out of the wound, which will then heal by first intention without inflammation and without "inflammatory" fever.

In making this statement I am not aware that there is a certain amount of fever following various severe wounds within twenty-four hours, even when no suppuration occurs. This wound-fever, however, is transitory, not high, and entirely different from the prolonged condition of high temperature formerly observed nearly always after operations and injuries. The occurrence of this inflammatory, traumatic, surgical, or symptomatic fever, as it was formerly called, means that the patient has been subjected to the poisonous influence of putrefactive or suppurative germs.

We now know why it is that certain cases of suppuration are not circumscribed but diffused, so that the pus dissects up the fascias and muscels, and destroys with great rapidity the cellular tissue. This form of suppuration is due to a particular form of bacteria called the pus-forming "chain-coccus." Circumscribed abscesses, however, are due to one or more of the other pus-causing micro-organisms.

How much more intelligent is this explanation than the old one that diffuse abscesses depended upon some curious characteristic of the patient. It is a satisfaction to know that the two forms of abscess differ because they are the result of inoculation with different germs. It is practically a fact that wherever there is found a diffuse abscess there will be discovered the streptococcus pyogenes, which is the name of the chain-coccus above mentioned.

So also is it easy now to understand the formation of what the the old surgeons called "cold abscess," and to account for the difference in appearance of its puriform secretion from the pus of acute abscess. Careful search in the fluid coming from such cold abscesses reveals the presence of the bacillus of tuberculosis and proves that a cold abscess is not a true abscess, but a local lesion of the tubercular infection. Easy is it now to understand the similarity between the "cold abscess" of the cervical region and the "cold abscess" of the lung in a phthisical patient. Both of them are, in fact, simply the result of invasion of the tissues with the ubiquitous tubercle bacillus; and are not due to pus-forming bacteria.

Formerly it was common to speak of the scrofulous diathesis, and attempts were made to describe the characteristic appearance of the skin and hair pertaining to persons supposed to be of scrofulous tendencies. The attempt was unsuccessful and unsatisfactory. The reason is now clear because it is known that the brunette or blonde, old or young, may become infected with the tubercle bacillus. Since the condition depends upon whether one or other becomes infected with the ever present bacillus of tubercle, it is evident that there can be no distinctive diathesis.

It is more than probable that the cutaneous disease so long described as lupus vulgaris is simply a tubercular condition of the skin, and not a special disease of unknown causation.

The metastatic abscesses of pyæmia are clearly explained when the surgeon remembers that they are simply due to a softened blood clot, containing pus-forming germs, being carried through the circulation and lodged in some of the small capillaries.

A patient suffering with numerous boils upon his skin has often been a puzzle to his physician, who has in vain attempted to find some cause for the trouble in his general health alone. Had he known that every boil owed its origin to pus bacteria which had infected a sweat gland or hair follicle, the treatment would probably have been more efficacious. The suppuration is due to pus-germs, either lodged upon the surface of the skin from the exterior, or deposited from the current of blood by which they have been carried to the spot.

I have not taken time to go into a discussion of the methods by which the relationship of micro-organisms to surgical affections has been established; but the absolute necessity for every surgeon to be fully alive to the inestimable value of aseptic and antiseptic surgery had led me to make the foregoing statements as a sort of resume of the relation of the germ theory of disease to surgical practice. It is clearly the duty of every man who attempts to practice surgery to prevent, by every means in his power, the access of germs, whether of suppuration, putrefaction, erysipelas, tubercle, tetanus or any other disease to the wounds or body of a patient. This as we all know, can be done by absolute bacteriological cleanliness. It is best, however, not to rely solely upon absolute cleanliness, which is almost unattainable; but to secure further protection by the use of antiseptic solutions. I am fully of the opinion that chemical antiseptics would be needless, if absolute freedom from germs was easily obtained. When I know that even such an enthusiast as I, myself, is continually liable to forget or neglect some step in this direction, I feel that the additional security of chemical antiseptics is of great value. It is difficult to convince the majority of physicians, and even ourselves, that to touch during an operation a finger to a door knob, to an assistant's clothing, or to one's own body may be to vitiate the entire operation by introducing one or two microbic germs into the wound.

An illustration of how careful the various steps of an operation should be guarded is afforded by the appended rules, which I have adopted at the Woman's Hospital of Philadelphia for the guidance of assistants and nurses. If such rules were taught every medical student and every physician entering practice as earnestly as the paragraphs of the catechism are taught the Sunday School pupil (and they certainly ought to be so taught) the occurrenee of suppuration, hectic fever, septicæmia, pyæmia and surgical erysipelas would be practically unknown. Death then would seldom occur after surgical operations except from hæmorrhage, shock or exhaustion.

With this feeble plea, Mr. President and Members of the Society, I hope to create a realization of the necessity for knowledge and interest in the direction of bacteriology, for this is the foundation of Modern Surgery. There is, unfortunately, a good deal of abdominal work done under the name of antiseptic and aseptic surgery, because the simplest facts of bacteriology are unknown to the operator. I have taken the liberty of bringing here a number of culture tubes containing beautiful specimens of some of the more common and interesting bacteria. The slimy masses seen on the surface of the jelly, contained in the tubes, are many millions of individual plants, which have aggregated themselves in various forms, as they have been developed as the progeny of the parent cells planted in the jelly as a nutrient medium or soil.

